# Psoriasis

**Published:** 1885-01

**Authors:** Henry J. Reynolds

**Affiliations:** Professor of Dermatology; 2200 Michigan ave.


### ARTICLE II.

PSORIASIS. *A Clinic at the College of Physicians and Surgeons of Chicago. By* HENRY J. REYNOLDS, M. D., *Professor of Dermatology.*

This man, aged 40, otherwise healthy, comes to us with a history of having had an eruption on his body for twenty-two years. He exposes an arm, and we find the eruption most marked upon the back or external surface, particularly so at or near the olecranon. The first striking thing, we observe about it, is its peculiar dryness, and its scaly condition. Upon scratching these scaly patches a little, we find that they assume a white, shiny, silvery appearance. Now, this of itself always suggests psoriasis. I think there is no other disease of the skin that gives just this character of scales, and when once familiar with the appearance of which, a diagnosis of psoriasis might almost be based upon this one symptom alone.

But while this of itself might be sufficient to bet on, it would be a very bad rule to follow, for we have other characteristics that are fully as valuable diagnostic points as this, and while any one of them alone might be sufficient to satisfy the mind of the physician, a combination of all is better and is the proper rule to follow in making a diagnosis of anything. Do not base the diagnosis upon one well marked symptom alone, but, hur riedly at least, run over the combination before committing yourself. Well, we now find upon removing the scaly patch, that we have what would scarcely have been expected from the mild, inactive appearance of the parts before this removal, a very red patch, and in this red patch we notice small pin-head-sized droplets of blood oozing out. Now this of itself again is almost pathognomonic of psoriasis. We further notice that the patches are round, not elevated, with well defined margins, and that the skin between them is perfectly normal. Now you will recollect that I have told you that you should always have an accurate knowledge of the condition of the skin of the entire body in diagnosticating skin affections, either from the statement of the patient or from personal examination (very preferably the latter). We find here and there, all over the body, the eruption more or less symmetrically distributed, with, however, a preference for the extensor surfaces. The patient states that he is generally freer from the disease in the summer than in the winter, and that it gives rise to no itching or other subjective sensation of any consequence. These symptoms are also characteristic of psoriasis. Now from the combination of symptoms just considered, viz.: the white, shiny scales, the dry character of the eruption, the round, isolated, red, bleeding patch on removal of the scales, its involving the entire body more or less, its preference for the extensor surfaces. its tendency to disappear in the warm and return in the cold weather, and the absence of itching, which embraces almost everything that is characteristic of the disease, we may with absolute positiveness render a diagnosis of psoriasis. Now some of these lesions on the trunk, from destruction of the scales by frequent washing and previous treatment, resemble the lesions of the macular syphiloderm, and he indeed states that he has been treated for secondary syphilis; but, if the man had syphilis half a dozen times, (which would be impossible) we know from the combination of symptoms in the case that he has psoriasis nevertheless; besides, he gives no syphilitic history, nor any of the numerous syphilitic symptoms more than that just referred to.

The eruption can very speedily be cleared up by appropriate treatment, but the great trouble in this disease is its tendency to return. We will first, for the purpose of getting rid of the scales, apply carbolized ointment twice a day for two days, then the entire body will be bathed in hot water with plenty of soap and friction. The same ointment will again be applied for two more days and followed by the same bathing process. We will then have a chrysarobin ointment, of grs. xx to Ung. Aquæ Rosæ ℥i, applied twice a day to every individual lesion on the body. The chrysarobin should be suspended and the former process substituted about every week or ten days. Arsenic may also be given internally in this disease, beginning with about five drops of Fowler's solution, three times a day, and gradually increasing as indicated by the effects.

For the prevention of the return of the disease frequent bathing with hot water and green soap, and measures which tend to keep the skin active, are indicated.

2200 Michigan ave.

